# Neuroblastoma by Stage at Diagnosis and Other Prognostic Factors: The Results of the BENCHISTA Italian Project—A Population-Based Study

**DOI:** 10.3390/cancers18040613

**Published:** 2026-02-13

**Authors:** Gemma Gatta, Riccardo Capocaccia, Massimo Conte, Marcella Sessa, Fabio Savoia, Carlotta Sacerdote, Walter Mazzucco, Rosalia Ragusa, Fabio Didonè, Laura Botta, BENCHISTA-Ita Working Group

**Affiliations:** 1 Evaluative Epidemiology Unit, Department of Epidemiology and Data Science, Fondazione IRCCS Istituto Nazionale Dei Tumori, 20133 Milan, Italy; fabio.didone@istitutotumori.mi.it (F.D.);; 2 Department of Statistics and Quantitative Methods, University of Milano-Bicocca, 20148 Milan, Italy; 3 Epidemiologia & Prevenzione, Indipendent Advisory Board, 20133 Milan, Italy; capocaccia.riccardo@gmail.com; 4 Clinical Oncology Unit, IRCCS Istituto Giannina Gaslini, 16147 Genoa, Italy; massimoconte@gaslini.org (M.C.); m.sessa@santobonopausilipon.it (M.S.); 5 Childhood Cancer Registry of Campania, AORN Santobono-Pausilipon, 80129 Naples, Italy; savoia@santobonopausilipon.it; 6 Unit of Cancer Epidemiology, Città della Salute e della Scienza University-Hospital and Center for Cancer Prevention (CPO), 10126 Turin, Italy; carlotta.sacerdote@cpo.it; 7 Department of Health Promotion, Mother and Child Care, Internal Medicine and Medical Specialties “G. D’Alessandro”, University of Palermo, 90127 Palermo, Italy; walter.mazzucco@unipa.it; 8 Division of Biostatistics and Epidemiology, Cincinnati Children’s Hospital Medical Centre, Cincinnati, OH 45229, USA; 9 Department of Paediatrics, University of Cincinnati College of Medicine, Cincinnati, OH 45229, USA; 10 Catania-Messina-Enna CR, Azienda Ospedaliero Universitaria Policlinico, 95123 Catania, Italy

**Keywords:** neuroblastoma, stage at diagnosis, survival, population-based cancer registry, children

## Abstract

**Background:** Neuroblastoma is the most common embryonal cancer. Since several population-based studies in Europe have revealed differences in 5-year survival across countries and regions. **Objectives:** We principally investigated the impact of stage at diagnosis on survival in Italy. **Methods:** Data were obtained from 26 population-based cancer registries (PBCRs), covering 319 cases (ages 0–14) diagnosed between 2013 and 2017, representing about 80% of the national population. Stage was classified according to the Toronto guidelines. Information on treatment and treating hospitals was also collected. Stage at diagnosis was further refined using probabilistic linkage with the Italian Neuroblastoma Registry (RINB). **Results:** Overall survival (OS), defined as all-cause mortality, was estimated using the Kaplan–Meier method. Most patients presented with stage M disease (37%), while a small proportion remained unclassified (2%). Three-year survival analysis showed significant differences between stages, ranging from >95% in localised (stage I) and MS stages to 78% in stage M (stage IV). No significant disparity across the Italian regions was observed in stage distribution or survival. Surgery and radiotherapy treatments of neuroblastoma were mostly centralized in the Centre and North of Italy. However, the cross-regional health migration from the South was limited to, and interpreted as appropriate for, high-volume centres. **Conclusions:** This population-based study highlights the high quality of care provided to children with neuroblastoma by an effective national clinical network. Compared with previous studies, we observed very limited variation across regions. A national childhood cancer registry remains essential to obtain a comprehensive picture of the country.

## Simple Summary

This population-based study aims to interpret possible survival differences for neuroblastoma in Italy. We investigate the impact of stage at diagnosis using the Toronto guidelines to standardise the classification of stage. This study, covering the 80% of the childhood population, reports high survival rates for children with neuroblastoma in Italy, with survival exceeding 95% in localized and MS stages and decreasing to about 78% in metastatic stage IV disease. No significant differences were observed across Italian regions in terms of stage at diagnosis or survival outcomes, indicating a high level of equity in care nationwide. Surgical and radiotherapy treatments were mainly centralized in high-volume centers located in the Centre and North of Italy. Patient migration from Southern regions was limited and appeared appropriate, reflecting referral to specialized centers. Overall, the findings highlight the effectiveness of the national clinical network in providing high-quality, standardized care for neuroblastoma across the country. Compared with earlier studies, regional variability completely reduced. The study also emphasizes the continued importance of maintaining a population-based childhood cancer registry, possibly increasing the population coverage versus a national childhood cancer registry, to ensure comprehensive monitoring of outcomes and to support ongoing improvements in paediatric oncology care.

## 1. Introduction

Neuroblastoma (NB), like all childhood cancers, is a rare tumour, but it is the most common among embryonal tumours. In Europe, slightly fewer than 900 new cases are diagnosed each year [1]. The highest incidence occurs in infants, and nearly all cases are diagnosed in children under 5 years of age [2]. In Europe, children with neuroblastoma have a 5-year survival rate of approximately 75%, with a similar proportion considered cured [3]. Age at diagnosis is a strong prognostic factor: 5-year survival at 1 year of age is about 90%, but it decreases to less than 60% in older children [3]. Survival disparities have been reported across European countries [3], ranging from 80% in Northern Europe to 62% in Eastern Europe, with Italy reporting 76%. Furthermore, survival is slightly higher in girls than in boys [2].

Alongside age and sex, stage at diagnosis is one of the major factors influencing outcome. Following the international study from the BENCHISTA project [4], the distribution of stage at diagnosis can now be described in a standardized way, according to the Toronto Guidelines (TG) [5], in cases codified and registered by population-based registries (PBCRs). For childhood cancers, these recommendations provide rules to improve data collection and coding of stage together with other prognostic factors [5]. The Italian counterpart of the BENCHISTA initiative [6] analysed survival by stage, geographic area, and other prognostic factors included in the TG. Hospitalization for diagnosis and treatment, as well as health migration, was also investigated. This observational study presents the results for neuroblastoma survival by stage in Italy, using data provided by PBCRs.

## 2. Materials and Methods

This observational study aimed at assessing stage distribution and survival outcomes for neuroblastoma in children across Italian population-based cancer registries (PBCRs), using standardized staging criteria and harmonized data collection methods [5,6].

Eligible cases included all children under 15 years of age diagnosed with neuroblastoma, between 1 January 2013 and 31 December 2017, in Italy. Cases were identified according to the ICCC-3 (International Classification of Childhood Cancer, 3rd edition) [7] as “category 4a: Neuroblastoma and Ganglioneuroblastoma”, combining morphology codes 9490 and 9500 with topography codes C00.0–C69.9, C73.9–C76.8, and C80.9 of the International Classification of Diseases in Oncology (ICD-O). Participating registries had to include at least three consecutive years of diagnosis and be followed up for three years.

A total of 319 cases of neuroblastoma were collected from 26 PBCRs accredited by the Italian Association of Cancer Registries (AIRTUM), covering 16 Italian regions. Data were enriched using probabilistic linkage with the Italian Neuroblastoma Registry (RINB) [1,8], which has allowed us to increase our database by 25 new cases.

Tumour stage at diagnosis was assigned using the Toronto Guidelines (TG) (Supplement Material Table S1). Staging followed the International Neuroblastoma Risk Group Staging System (INRGSS), with stage assigned at diagnosis, prior to any surgical resection. In specific cases, post-surgical imaging to rule out metastases was accepted if performed before systemic therapy. For neuroblastoma, the TG define two staging tiers: Tier 2 is equivalent to the International Neuroblastoma Risk Group Staging System (INRGSS) [5,9], and Tier 1 is a simplified version thereof, applicable when cross-sectional imaging information is not available.

To ensure consistent application of the TG, the BENCHISTA project implemented a structured training program, including in-person workshops (e.g., Palermo, February 2020) and online sessions led by clinical experts (Spring 2023), involving cancer registry staff and paediatric oncologists. Staging data quality was supported through case-based exercises and a dedicated helpdesk [10].

Italian regions were categorized into three macro-areas: North, Centre, and South–Islands (South) (Table 1).

## 3. Statistical Analysis

Descriptive statistics were used to report the distribution of cancer stages and geographic areas. Overall survival (OS), defined as all-cause mortality, was preferred to cause-specific relative survival due to the uncertainties in attributing cause of death and to the negligible mortality rates in Italian children. OS was estimated using the Kaplan–Meier method to generate non-parametric survival curves with 95% confidence intervals, stratified by geographical area and stage. Differences in survival distributions between stages and regions were assessed using the log-rank test. Multivariable survival analysis was carried out by the Cox model. To analyse patient care pathways, we examined the proportion of cases diagnosed and treated within the same region.

The formal statistical power to detect geographical differences in stage distribution and survival is necessarily limited by the rarity of the disease. Approximately, we had 70% power to detect a 10% difference in survival between two regional groupings with 150 cases in each group, or a 12.5% difference for group size with 100 cases each.

Ethical Approval and GDPR Compliance: Ethical approval for the project has been given by the Ethical Committee of the Fondazione IRCCS “Istituto Nazionale dei Tumori” (INT) during the e-session on 25 May 2021. The format of the data items to be collected has been agreed with the registries to be in the maximally de-identified format that minimises any risks to data privacy, in compliance with GDPR. INT will act as Data Controllers for the project, with responsibilities as defined by article 26, GDPR, and with the legal basis for data processing under article 6, GDPR 2018, being “Public interest” (clause (e)) and article 9 (special category) clause (j)—“research”.

## 4. Results

Out of a total of 319 cases, 43% were from Northern Italy, 34% from the South, and 23% from the Central regions (Table 1).

According to the detailed stage classification (Tier 2), most patients were diagnosed with stage M disease (37%), followed by L2 (27%), L1 (24%), and MS (10%), with a small proportion (2%) of unclassifiable cases (X) (Table 2). No significant variability in stage distribution was observed between North, Centre, and South/Island (South), although both the North and South showed a higher proportion of metastatic NB compared to the Centre.

**Figure 1:**
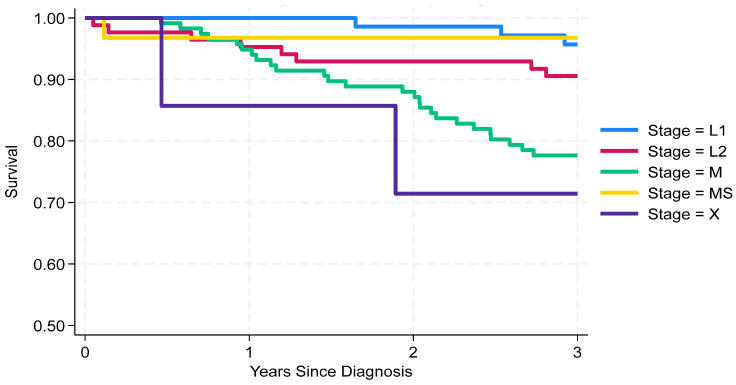
Neuroblastoma in Italy: 3-year survival by stage (tier 2 Toronto guidelines).

The 3-year survival analysis revealed significant differences across stages. Patients in stage MS (97%, 95% CI: 79–99%) and L1 (96%, 95% CI: 87–99%) had the highest survival, followed by L2 (91%, 95% CI: 82–95%) and M (78%, 95% CI: 69–84%). Stage X showed the lowest survival rate (71%, 95% CI: 26–92%), although the number of cases was limited (Figure 1). These findings confirm the importance of early staging and accurate classification for prognosis.

**Figure 2:**
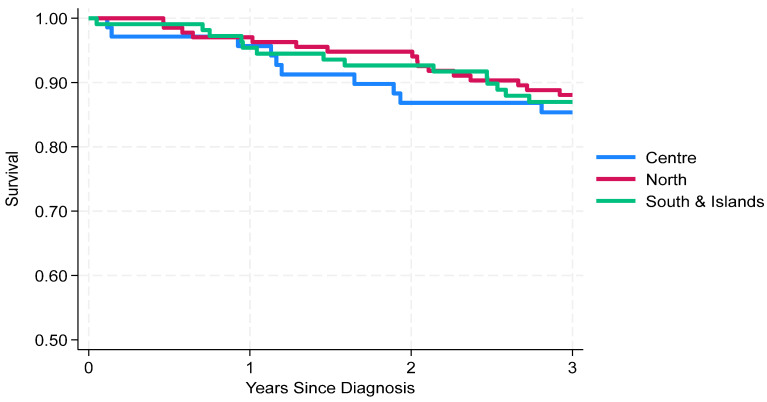
Neuroblastoma in Italy: 3-year survival by area.

From a geographical perspective, the 3-year OS was similar across areas (Figure 2): 88% (95% CI: 81–93%) in the North, 87% (95% CI: 79–92%) in the South, and 85% (95% CI: 75–92%) in the Centre.

We estimated geographical differences, adjusting by age, stage, and N-myc, by applying the Cox model (Supplement Table S2). The results of the univariate analysis were confirmed: no significant differences across areas were found after adjustment while significantly higher risks were reported for increasing ages, more advanced stage at diagnosis, and N-myc expression.

Considering primary treatment, almost all children (93%) underwent surgery regardless of stage. L1 patients were treated exclusively with surgery. Nearly all L2 patients received both surgery and chemotherapy, and less than one third also had radiotherapy. M patients were managed similarly to L2, but 66% additionally received radiotherapy. MS patients were almost all treated surgically, and about half also underwent chemotherapy (Table 3). An important aspect of the analysis focused on interregional mobility (Table 4) during diagnosis and treatment.

According to hospital data, 89% of patients were diagnosed in a hospital within their region of residence, while 8% received diagnosis outside their region (for 3% of cases, hospital of diagnosis was not available). In total, 77% of surgical treatment was provided within the region, while 18% of patients migrated outside. Chemotherapy was administered locally for 84% of patients, with 13% treated elsewhere. Radiotherapy showed the highest mobility: only 65% were treated in their region, while 27% received treatment outside. Patients residing in the South were those who most frequently migrated for hospitalization, particularly for treatment. Notably, only 33% of patients from the South underwent radiotherapy in their region of residence, and 67% received surgery locally. Survival was slightly but not significantly different between patients treated in the same area of residence and those who migrated to a different area. Once adjusted by a multivariable Cox regression analysis, the estimated relative risk of death of migrated with respect to non-migrated patients was 1.15 (C.I. 0.47–2.82) as shown in Supplemental Table S3.

## 5. Discussion

In Italy, the 3-year survival for NB cases was 86%, between 2013 and 2017, representing one of the best outcomes compared with other European regions of the BENCHISTA international study [11]. Survival figures were consistent with stage at diagnosis: poorest in children with distant metastases and highest in those with localized (Stage I) and special metastatic (MS) stages, as reported in other population-based studies [12,13]. Despite minor geographical discrepancies in the proportion of metastatic disease at diagnosis, the three macro-areas analysed did not show differences in survival after three years.

According to the earlier monographic report on childhood cancers in Italy [14], during 2003–2008, 3-year survival for neuroblastoma in the South was slightly below 70%, with the other regions ranging between 80% and 90%. More recently, the AIRTUM-supported study [15] reported no differences in the 5-year regional survivals, evidence consistent with our findings, which were further enriched by a detailed description of stage at diagnosis. With our study, we can conclude that the gap between North/Centre and South has been closed. Actually, both distribution by stage at diagnosis and 3-year survival across areas were very close. There are other prognostic factors that one can consider: age, sex and N-myc amplification were all distributed in a similar way in the three areas (see Table 1).

According to the international BENCHISTA study [16], the Italian distribution by stage showed slightly fewer L1 cases (24% vs. 32%) and slightly more metastatic (M) cases (37% vs. 34%) compared with the BENCHISTA Southern European region, where Italy is included. However, the 3-year survival figures in Italy were similar to those reported internationally, both in Southern and Northern Europe (85%) [11]. Eight cases were reported by cancer registries with unknow stage at diagnosis. They were not recruited by the NB national clinical registry. We suppose that these cases skipped the NB network because they represented aggressive diseases and were not able to reach the hospital of excellence in time. This suggests that clinical-based survival, differently from population-based survival, might be biased because of potentially losing some bad prognosis cases.

As expected, all the children affected by NB were surgically treated and the majority of them also received chemotherapy. Our information on treatments is not precise. Actually, we cannot provide a more detailed description of the treatments protocol provided; that was outside the aims of the study.

This study highlights a high percentage of in-region diagnoses, reflecting good territorial coverage for initial assessments. Slightly lower in-region percentages for surgery and radiotherapy underline the importance of centralization in hospitals with high case volumes. The slightly higher proportion of migration was from the South, advanced cases (L3 and M) that moved to the North and Centre of the country. Although high-volume centres were more common in Northern and Central Italy, outcomes did not appear to be affected by the residence of the case. However, migration was not significantly associated with worse survival. This is a very positive result provided by a population-based study on childhood cancers, highlighting the efficiency of the Italian national health system, supported by primary care paediatricians and the Italian Association for Pediatric Hematology and Oncology (AIEOP) specialised clinical network [17].

The findings of the present study are relevant on a global scale, since they contribute to the larger international BENCHISTA study, in which more than 70 registries in 24 countries centralised data with the aim of providing an interpretation of outcome disparities. This collaboration has shown that, even if Italy is among the countries with the best outcomes for NB, there is still room for future improvement.

## 6. Limitations

The study period 2013–2017 is not as recent as one may expect; however, the length of the procedure to collect all the necessary information, in part due to the application of the GDPR, only allowed us to study the period 2013–2017 and assure a follow-up of 3 years. We can notice that, after the study period, there were no important improvements in therapeutic protocols: survival was only unfavourable in metastatic cases, for which no progress was reported in the literature.

Furthermore, disease-free survival would be informative and useful to compare with clinical studies, but this data was available only for 68% of cases and we hope to complete it, together with a longer follow-up, in the second phase of the Benchista project [12].

Finally, despite our study being based on a sample from about 80% of the national population, we are aware that regions not included in the analyses are those usually facing significant challenges in childhood cancer care, as reflected by the lower number of AIEOP centers in network for these regions [18]. Therefore, a national childhood cancer registry remains essential to obtain a comprehensive picture of the country [19]. Actually, we believe that population-based findings can inform future policy, resource allocation, and registry development at national level.

## 7. Conclusions

In conclusion, we documented a survival improvement. For the first time, we provide population-level survival estimates at 3 years by stage and region for NB. This achievement was made possible by the adoption of the Toronto Guidelines and investment in cancer registration to collect clinical variables.

## Figures and Tables

**Table 1:** Neuroblastoma in Italy: number of cases, mean age, male/female ratio, quality of cancer site codification, loss to follow-up and N-myc expression by geographical area. Period of diagnosis: 2013–2017.

Geographical Area	N	%	Mean Age at Diagnosis in Months	M/F Ratio	Lost to Follow-Up/Censored	Unknown Primary Site (%)	Positive N-Myc § (%)
Centre *	74	23.2	27.2	0.90	8%	0	22%
North ^	136	42.6	30.9	1.19	2%	0	30%
South and Islands °	109	34.2	27.6	0.91	4%	0	28%
Total	319	100.0	28.9	1.02	4%	0	27%

* = Marche, Toscana, Umbria, Lazio; ^ = Bergamo, Insubria Varese Como, Mantova e Cremona, Emilia Romagna, Friuli Venezia Giulia, Genova, Milano, Monza e Brianza, Piemonte, Trento, Veneto; ° = Puglia, Basilicata, Catania–Messina–Siracusa–Enna, North of Sardinia, Nuoro, Palermo, Ragusa and Caltanisetta, Siracusa, Trapani, Campania; § = based on the cases with N-Myc performed.

**Table 2:** Neuroblastoma in Italy: distribution by stage at diagnosis according to tier 2 Toronto guidelines.

Neuroblastoma Stage Tier 2						
Area	L1	L2	M	MS	X	Total
Centre Number	19	23	21	7	4	74
%	25.7	31.1	28.4	9.5	5.4	100
North Number	31	32	55	14	4	136
%	22.8	23.5	40.4	10.3	2.9	100
South and Islands Number	27	30	42	10	0	109
%	24.8	27.5	38.5	9.2	0	100
Total Number	77	85	118	31	8	319
%	24.1	26.7	37	9.7	2.5	100

**Table 3:** Neuroblastoma in Italy: distribution of treatment by stage at diagnosis.

Treatments	TG Stage at Diagnosis (T2)					
	L1	L2	M	MS	X	Total
Chemotherapy						
No	82%	7%	0%	39%	25%	26%
Yes	12%	92%	97%	58%	50%	70%
Unknown	6%	1%	3%	3%	25%	4%
	100%	100%	100%	100%	100%	100%
Surgery						
No	1%	1%	9%	3%	13%	5%
Yes	96%	99%	88%	94%	75%	93%
Unknown	3%	0%	3%	3%	13%	2%
	100%	100%	100%	100%	100%	100%
Radiotherapy						
No	92%	64%	30%	97%	50%	61%
Yes	1%	28%	66%	0%	0%	32%
Unknown	6%	8%	4%	3%	50%	7%
	100%	100%	100%	100%	100%	100%

**Table 4:** Benchista in Italy: hospitals of diagnosis and treatment by region and area of residence.

		Diagnosis %	Surgery %	Chemo %	Radio %
Area/Region	No				
NORTH	136	91	88	88	78
Veneto	23	83	94	95	75
Lombardy	44	100	97	97	93
Friuli	8	100	0	80	100
Trentino	3	33	0	0	0
Genoa	5	100	100	100	100
Emilia Romagna	6	83	67	0	0
Piedmont	47	89	82	83	64
CENTRE	74	89	71	92	25
Umbria	6	17	67	50	unknown
Marche	14	93	75	91	33
Tuscany	20	90	67	100	0
Lazio	34	100	100	97	100
SOUTH and ISLANDS	109	78	62	77	50
Campania	31	84	71	92	57
Apulia	35	83	48	57	14
Basilicata	3	0	0	0	0
North of Sardinia	4	25	0	0	0
Sicily	36	83	69	89	78

## Data Availability

The original contributions presented in this study are included in the article/Supplementary Material. Further inquiries can be directed to the corresponding author.

## Supplementary Materials

The following supporting information can be downloaded at https://www.mdpi.com/article/10.3390/cancers18040613/s1, Table S1: Toronto Guidelines staging criteria for neuroblastoma, Table S2: Neuroblastoma in It-aly : adjusted hazard ratio for age, region, stage at diagnosis and N-myc expression, Table S3: Neuroblastoma in Italy: adjusted hazard ratio for age, region, stage at diagnosis and N-myc ex-pression and migration for treatment.

## Appendix A

BENCHISTA-Ita WG: Basilicata Cancer Registry, IRCCS CROB: Rocco Galasso. Bergamo Cancer Registry: Giuseppe Sampietro. Campania Childhood Cancer Registry: Marcella Sessa*, Patrizia Piga. Childhood Cancer Registry of Piedmont: Milena M Maule, Carlotta Sacerdote*. Cremona & Mantova Cancer Registry: Paola Ballotari. Friuli Venezia Giulia Cancer Registry, CRO Aviano National Cancer Institute: Luigino Dal Maso Integrated Cancer Registry CT-ME-EN: Rosalia Ragusa, Antonina Torrisi. Liguria Cancer Registry, Ospedale Policlinico San Martino IRCCS: Luca Boni. Monza-Brianza Cancer Registry: Magda Rognoni. Palermo Province Cancer Registry: Rosalba Amodio. Puglia Cancer Registry: Francesco Cuccaro, Danila Bruno. Registro Tumori ATS Della Città metropolitana di Milano: Antonio G Russo, Federico Gervasi. Registro Tumori ATS Insubria: Maria L Gambino. Registro Tumori dell’Emilia-Romagna, Unità di Piacenza: Elisabetta Borciani. Registro Tumori dell’Emilia-Romagna, Unità di Parma: Maria Michiara. Registro Tumori dell’Emilia-Romagna, Unità di Reggio Emilia: Lucia Mangone. Registro Tumori dell’Emilia-Romagna, Unità di Modena: Gianbattista Spagnoli. Registro Tumori dell’Emilia-Romagna, Unità di Ferrara: Stefano Ferretti. Registro Tumori dell’Emilia-Romagna, Unità Della Romagna, IRCCS IRST Meldola: Silvia Mancini. Registro Tumori di Ragusa e Caltanissetta: Eugenia Spata. Registro Tumori Regione Marche: Sonia Manasse, Paola Coccia. Registro Tumori Umbria: Fabrizio Stracci Registro Tumori Sassari: Daniela Piras Registro Tumori Lazio: Ilaria Cozzi. Registro Tumori Nuoro: Pasquala Pinna Siracusa Cancer Registry: Francesca Bella. Toscana Cancer Registry: Adele Caldarella, Teresa Intrieri. Registro Tumori Trapani_Agrigento, Dipartimento di Prevenzione, ASP Trapani: Tiziana Scuderi. Trento Cancer Registry (Trento CR), Servizio Epidemiologia Clinica e Valutativa, APSS Trento: William Mantovani. Veneto Cancer Registry, Regional Epidemiological Service, Azienda Zero, Padova, Italy: Manuel Zorzi, Stefano Guzzinati.

Project Management Team (PMT): Riccardo Capocaccia, Epidemiologia e Prevenzione, Indipendent Advisory Board, Milan, Italy; Massimo Conte, Clinical Oncology Unit, IRCCS Istituto Giannina Gaslini, Genoa, Italy; Fabio Savoia, Campania Childhood Cancer Registry, Naples, Italy.

Clinical Lead Experts/Other Representatives: Andrea Di Cataldo: Università di Catania, Catania, Italy; Paolo D’Angelo: Ospedali Civico di Cristina Benfratelli di Palermo; Andrea Pession: IRCCS Azienda Ospedaliero-Universitaria di Bologna, Bologna, Italy; Riccardo Haupt, Maria Luisa Garrè: IRCCS Istituto Giannina Gaslini, Genoa; Gianni Virgili: University of Florence, Florence, Italy; Andrea Tittarelli, Claudio Tresoldi, Giovanna Tagliabue: Department of Predictive Medicine and Prevention at the National Cancer Institute of Milan, Italy.* PMT member.
